# Artificial intelligence risk prediction model for common respiratory pathogens in China based on heterogeneous multi-source clinical and geographic data: A modeling study

**DOI:** 10.1371/journal.pdig.0001553

**Published:** 2026-07-21

**Authors:** Hongyu Wang, Qianqian Zhang, Binhuang Sun, Dan Shen, Lingling Lu, Huan Li, Kai Fang, Hongyang Li, Hui Yan, Feiying Chen, Tingting Zhao, Ling Chen, Mengyu Rong, Wen Liu, Zengyun Hu, Jingwen Ai, Wenhong Zhang

**Affiliations:** 1 Department of Infectious Diseases, Shanghai Key Laboratory of Infectious Diseases and Biosafety Emergency Response, National Medical Center for Infectious Diseases, Huashan Hospital, Fudan University, Shanghai, China; 2 School of Public Health, Shanghai Jiao Tong University School of Medicine, Shanghai, China; 3 Key Laboratory of Digital Technology in Medical Diagnostics of Zhejiang Province, Dian Diagnostics Group Co., Ltd., Hangzhou, China; 4 Infection Technology Platform, Dian Diagnostics Group Co., Ltd., Hangzhou, China; 5 Sales & Marketing Center, Dian Diagnostics Group Co., Ltd., Hangzhou, China; 6 Shanghai Sci-Tech Inno Center for Infection & Immunity, Shanghai, China; 7 National Clinical Research Center for Aging and Medicine, Huashan Hospital, Fudan University, Shanghai, China; 8 Key Laboratory of Medical Molecular Virology (MOE/MOH), Shanghai Medical College, Fudan University, Shanghai, China; Hadassah Academic College, ISRAEL

## Abstract

Most respiratory pathogens exhibit distinct seasonal and periodic outbreak patterns driven by climatic factors. However, predictive models that jointly consider climate, air quality index (AQI), and socioeconomic variables are lacking. We retrospectively analyzed targeted or metagenomic next-generation sequencing data from 153,544 respiratory samples collected from 1,880 centers across 30 provinces in China between September 2022 and September 2024. Monthly positivity rates were matched with geographic, climatic, AQI, and GDP data. CO(0.098 ± 0.016), HCHO(0.096 ± 0.021), O3(0.102 ± 0.019), sunshine hours(0.103 ± 0.028), wind speed(0.114 ± 0.024), and GDP(0.095 ± 0.019). were identified as the key geographical factors for the positivity across most respiratory pathogens via mean Gini index reduction, and a gradient boosting decision tree(GBDT) model was trained and benchmarked against other AI methods using the DISO metric. This model accurately simulated the epidemiological trends from September 2022 to September 2024 and outperformed alternative models with the lowest DISO metric of 0.12 in influenza A, 0.21 in SARS-CoV-2, 0.25 in RSV. The GBDT model was used to predict the short-term epidemic of 10 respiratory pathogens between October and December 2024. The predictions showed consistent trends with the external validation cohort for RNA viruses including SARS-CoV-2 and influenza A virus, but differed for bacterial pathogens. Integrating air quality, climatic, and socioeconomic data yields robust predictions of respiratory infection dynamics in the short-term by the GBDT model, bolstering public health surveillance and offering a framework potentially applicable to other infectious diseases.

## Author summary

Numerous studies have explored the links between respiratory infections and factors like air quality, economic conditions, and climate. However, there is still limited evidence on how these factors collectively affect the positivity rates of various respiratory pathogens, and no effective predictive models exist. Our study analyzed over 150,000 respiratory infection samples collected in China from September 2022 to September 2024, linking them with data on air quality, weather, and local economic conditions. We found that pathogen detection rates were correlated with geographical factors including CO (0.098 ± 0.016), HCHO (0.096 ± 0.021), O3 (0.102 ± 0.019), sunshine hours (0.103 ± 0.028), wind speed (0.114 ± 0.024), and GDP (0.095 ± 0.019). Using these insights, we developed a predictive model on national surveillance data, accurately forecasting coronavirus and influenza infections in multiple regions. This model outperformed other common tools, with the lowest DISO metrics of 0.12 in influenza A, 0.21 in SARS-CoV-2, 0.25 in RSV, showing potential for broader use. Our study highlights the need for early warning systems and provides a reliable method to predict future pathogen trends, helping public health officials respond more effectively.

## Introduction

Respiratory infections remain the most prevalent type of infection globally, with 12.8 billion upper respiratory infections reported in 2021 (162,484·8 cases per 10,000 population) and lower respiratory infections among the top three global killers [1]. Their marked seasonality, diverse pathogens and rapid spread—exemplified since 2019 by alternating SARS-CoV-2 and influenza waves alongside *Mycoplasma pneumoniae*, rhinovirus, and adenovirus—have strained socioeconomic development and public health. Highly pathogenic and transmissible respiratory pathogens remain a key focus of surveillance for healthcare systems worldwide. However, conventional surveillance, reliant on case reporting, invariably lags transmission.

China’s vast, climatically varied terrain from tropical monsoon to plateau climates offers a natural laboratory for studying climate-pathogen correlations in respiratory infections. A pre-COVID study analyzed pathogen spectrum data from 28,369 hospitalized cases of acute respiratory infections between 2009–2013 [2]. The study identified temperature, vapor pressure, atmospheric pressure, rainfall, and sunlight duration as primary drivers of seasonal patterns for most viruses. However, since the COVID-19 pandemic, the implementation and subsequent lifting of non-pharmaceutical interventions (NPIs) have significantly altered the epidemiological dynamics of respiratory pathogens compared with pre-COVID patterns [3]. This shift necessitates a re-evaluation of climate-pathogen relationships. Transitioning from reactive, report-based monitoring to proactive systems that integrate environmental predictors—such as climate anomalies, genomic wastewater surveillance and real-time air sampling—is essential for timely early warning and effective control of respiratory infections.

Numerous studies have suggested that climatic and environmental factors can predict short-term variations in epidemic trajectories. Wang Xia et al. introduced the “Yihuajiemu” model to analyze COVID-19 incidence and transmission patterns climatically analogous regions in both hemispheres, projecting a prolonged pandemic phase with potential one- to two-year cyclical transmission modulated by climate [4]. Zhiyuan Chen et al. compared the epidemiological characteristics of influenza A virus before and after the COVID-19 pandemic, revealing that pandemic-related interventions reshaped the lineage distribution, transmission intensity, and seasonality of influenza viruses worldwide [5]. However, these studies largely focus on single-pathogen dynamics and offered limited insights into the interactive patterns of co-circulating respiratory pathogens (e.g., SARS-CoV-2, influenza, RSV, and *Mycoplasma pneumoniae*). The absence of frameworks to model multi-pathogen synergies or competition—particularly under shifting climate conditions and immunity landscapes—represents a critical gap in predicting respiratory disease burdens and optimizing public health strategies.

Pathogen positivity rate serves as a crucial indicator for tracking transmission trends and epidemic intensity of respiratory infectious diseases**,** particularly amid influenza, SARS-CoV-2, and respiratory syncytial virus (RSV) [6,7]. However, traditional statistical models have limitations when handling the high-dimensional, multi-source, nonlinear, and spatiotemporally heterogeneous data from hospitals and public-health laboratory networks to meteorological and other external variables [8–10]. To address these challenges, a growing body of research has adopted machine learning approaches for modeling and predicting pathogen positivity rates [11–13]. Algorithms such as random forests, support vector machines (SVM), and artificial neural networks (ANN) reveal how meteorological and environmental factors influence pathogen transmission, enabling short-term outbreak warnings, high-risk region identification, co-circulating pathogen prediction [14,15]. These advancements position machine learning as a pivotal tool in intelligent infectious-disease surveillance systems. However, no established technical methodology currently exists for integrating heterogeneous clinical and geographic data into multi-factor risk assessment and early warning.

Since 2022, the National Center for Infectious Diseases of China has established a three-tier sentinel surveillance network for respiratory pathogens across mainland China (approximately 4°N ~ 53°N), spanning tropical, temperate, and frigid zones within seven major geographical regions. We collected more than 150,000 respiratory targeted or metagenomic next-generation sequencing (tNGS or mNGS) test records (2022–2024) with paired clinical characteristics and test results alongside geographic coordinates, climate data, air quality indices, and regional development indicators. By combining correlation analysis with feature-importance ranking, we explored both linear and nonlinear relationships between respiratory infection prevalence and geographic characteristics across China’s diverse regions, then developed a GBDT model to predict short-term epidemic trends of common respiratory pathogens and compared consistency with 2 external cohorts.

## Results

### Study flow and demographic characteristics

**Fig 1:**
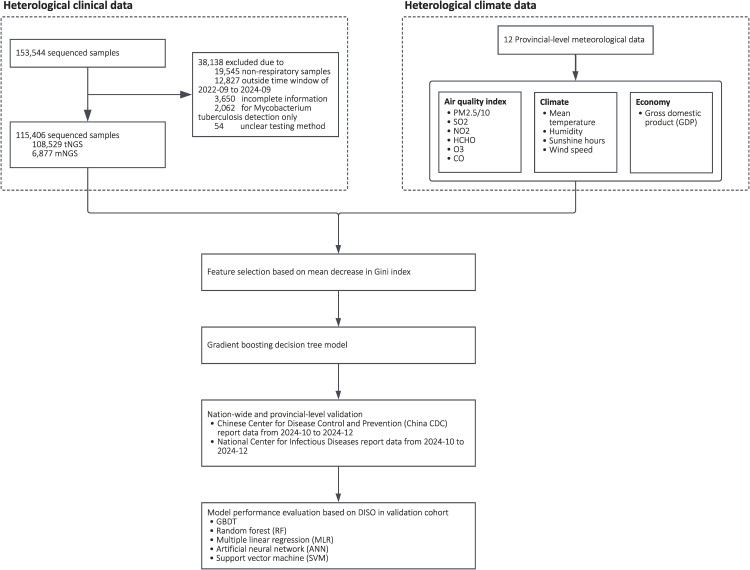
Study flowchart.

This retrospective cohort study initially collected 153,544 samples tested by mNGS or tNGS between September 2022 and September 2024. A total of 38,138 cases were excluded due to: non-respiratory sample types (19,545 cases), incomplete information (3,650 cases), exclusive detection of Mycobacterium tuberculosis (2,062 cases), unspecified testing methods (54 cases), and exceeding the time window (12,827 cases). Ultimately, 115,406 respiratory samples tested by mNGS or tNGS were included in the downstream analysis, comprising 6,877 mNGS-tested samples and 108,529 tNGS-tested samples (Figs 1 and S1 and S1 Table). Partial overlap existed among the detection methods, all of which could identify 49 pathogens, including 28 common respiratory pathogens mentioned above.

**Fig 2:**
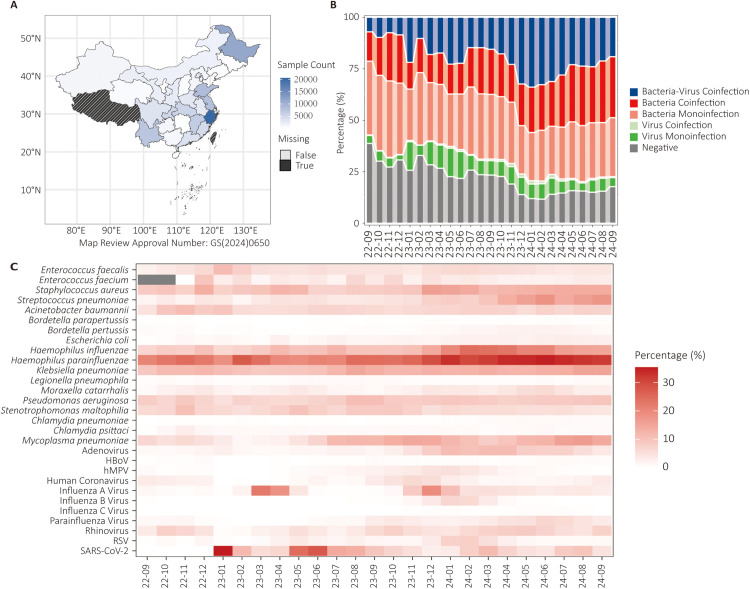
Spatiotemporal endemic patterns of common respiratory pathogens. **(A)** National sample distribution at provincial level. **(B)** Monthly positivity of common community-acquired respiratory pathogens nationwide. **(C)** temporal prevalence of coinfection patterns. Abbreviation: HBoV: Human Bocavirus, hMPV: Human Metapneumovirus, RSV: Respiratory Syncytial Virus.

Among the 115,406 included participants, the median age was 58 years (IQR 35–70). The cohort comprised 14,517 (12·58%) children aged 0–7 years, 4,621 (4·0%) children aged 8–14 years, 39,809 older adults aged 65–84 years, and 3,963 (3·43%) individuals over 85 years. Lower respiratory tract samples accounted for 104,719 cases (90·74%) (S1 Table). The 108,529 samples were collected from 1,880 centers across 30 provinces/municipalities in China. Zhejiang Province contributed the largest sample size (10,519 cases), while Ningxia Hui Autonomous Region had the smallest (114 cases) (Fig 2A).

Fig 2B and S1 Table illustrate the epidemiological patterns of common respiratory pathogens in China from September 2022 to September 2024. The highest detection rates were observed for *Haemophilus influenzae* (18,610 [16·13%]) and *Haemophilus parainfluenzae* (32,048 [27·77%]), followed by *Klebsiella pneumoniae* (14,741 [12·77%]), *Staphylococcus aureus* (14,074 [12·2%]), and *Mycoplasma pneumoniae* (13,083 [11·34%]). Among viruses, the top three were rhinovirus (6,356 [5·51%]), adenovirus (7,294 [6·32%]), and influenza A virus (6,102 [5·29%]). *Haemophilus influenzae* and *Haemophilus parainfluenzae* maintained persistently high detection rates throughout the two-year period, plateauing at peak levels in the first half of 2024. SARS-CoV-2 exhibited elevated detection rates in January 2023, May, and June 2023, likely corresponding to the first wave of BA.5 infections following China’s relaxation of COVID-19 control measures and a subsequent XBB variant wave. Influenza A virus showed atypical peaks in March and April 2023, deviating from its usual winter predominance, with resurgence observed in December 2023. *Mycoplasma pneumoniae* circulated prominently during autumn-winter seasons of 2023–2024, while adenovirus surged in autumn-winter 2023. These trends align with Chinese CDC surveillance reports, though no publicly available data exist for other pathogens.

The incidence of bacterial-viral co-infections exhibited marked seasonal fluctuations with an upward annual trend, demonstrating periodic surges during winter 2022, spring 2023, winter 2023, and early 2024 (Fig 2C).

### Correlation between respiratory pathogen endemics and geographic factors

Pearson correlation analysis was conducted to examine the relationships between climate, air quality, socioeconomic indicators, and positivity rates of common respiratory pathogens in China, as shown in S2 Fig. The results revealed that most air quality factors were positively correlated with the detection rates of common respiratory bacteria/viruses, while formaldehyde (HCHO) predominantly showed negative correlations. Temperature exhibited primarily negative correlations, whereas wind speed demonstrated positive correlations with bacterial infections but negative correlations with viral infections. GDP was negatively correlated with bacterial infections but positively correlated with most RNA viral infections (except for Influenza A and RSV). Regarding infection patterns, bacterial-viral co-infections were mainly associated with AQI, with temperature and humidity exerting greater influence on bacterial infections, while AQI had a more pronounced effect on viral infections (S2 Fig).

### Screening of key driving factors

**Fig 3:**
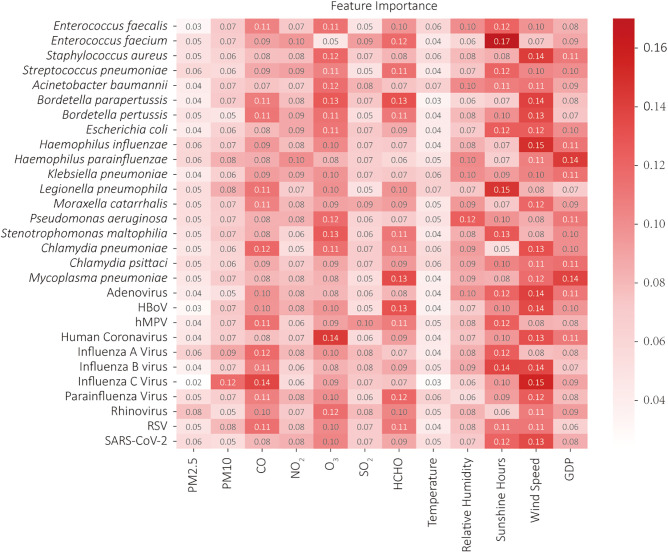
Feature importance of geographic factors on common respiratory pathogens nationwide. Abbreviation: HBoV: Human Bocavirus, hMPV: Human Metapneumovirus, RSV: Respiratory Syncytial Virus.

The nonlinear relationships between respiratory pathogen positivity rates and contributing factors were quantified using the feature importance method based on mean Gini index reduction, with driving factors exceeding 0.1 being selected as shown in Fig 3. The analysis identified CO (0.098 ± 0.016), HCHO (0.096 ± 0.021), O3 (0.102 ± 0.019), sunshine hours (0.103 ± 0.028), wind speed (0.114 ± 0.024), and GDP (0.095 ± 0.019) as key drivers of positivity rate variations for most common respiratory pathogens.

### National modeling of respiratory pathogen transmission dynamics

**Fig 4:**
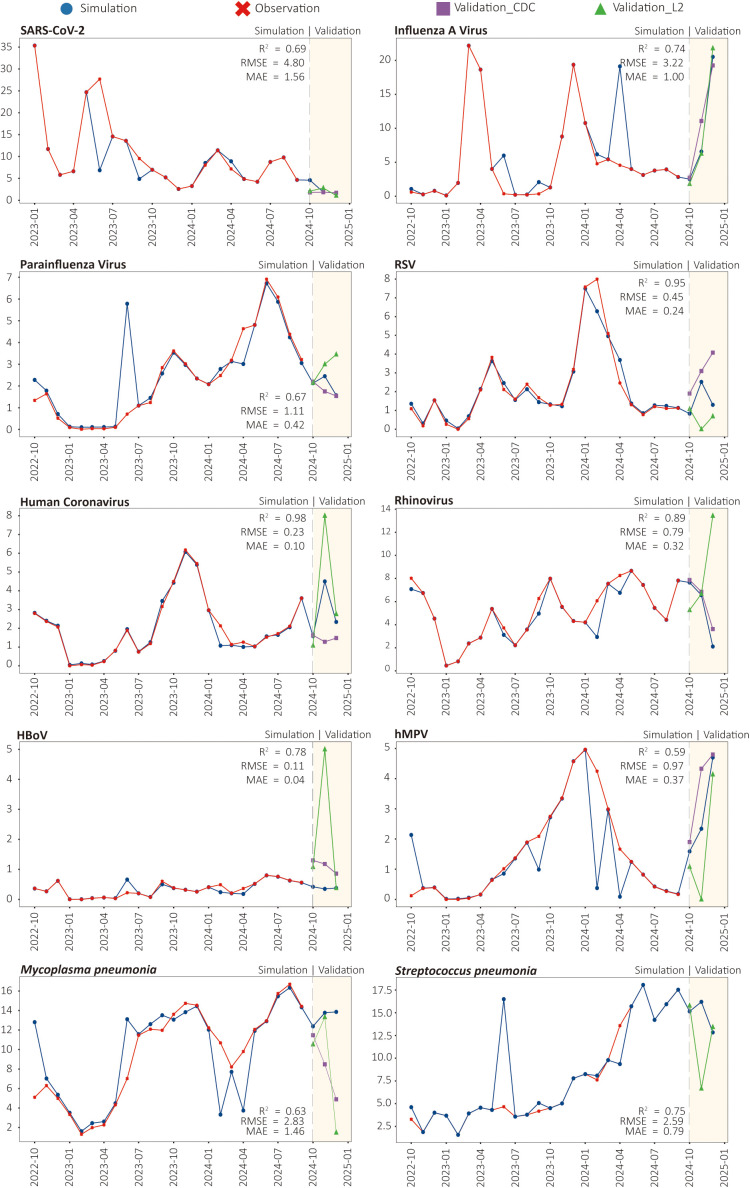
GBDT Modeling and external validation on 10 Respiratory Pathogen Positivity Rate at nationwide. Abbreviations: HBoV: Human Bocavirus, hMPV: Human Metapneumovirus, RSV: Respiratory Syncytial Virus, R2: Coefficient of Determination, RMSE: Root Mean Square Error, MAE: Mean Absolute Error.

We established a novel GBDT model based on the aforementioned key driver factors and simulated the epidemiological trends of ten common respiratory pathogens across China from September 2022 to September 2024 and compared the results with actual surveillance data (Fig 4). The model demonstrated strong concordance with real-world observations for influenza A virus (R² = 0.74), human coronaviruses (R² = 0·98), RSV (R² = 0·95), rhinovirus (R² = 0·89), and Streptococcus pneumoniae (R² = 0·75). However, the simulation showed relatively poorer consistency with actual data for SARS-CoV-2 (R² = 0·69), parainfluenza virus (R² = 0·67), and Mycoplasma pneumoniae (R² = 0·63).

### Regional modeling of respiratory pathogen transmission dynamics

**Fig 5:**
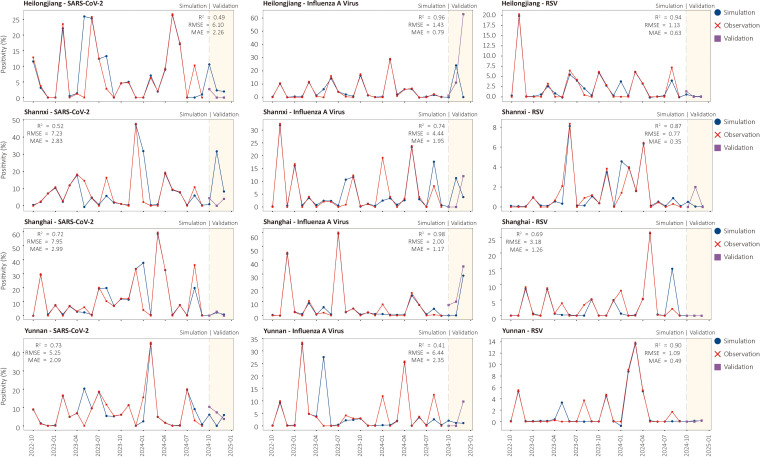
GBDT Modeling and external validation on SARS-CoV-2, Influenza A Virus, RSV Positivity Rate in Heilongjiang, Shannxi, Shanghai and Yunnan. Abbreviations: RSV: Respiratory Syncytial Virus, R2: Coefficient of Determination, RMSE: Root Mean Square Error, MAE: Mean Absolute Error.

Due to China’s vast territorial expanse, nationwide modeling approaches often fail to account for regional disparities in climate and socioeconomic development, resulting in predictive uncertainties. We therefore conducted spatial stratification based on data availability and geographical representativeness (selecting Heilongjiang, Shaanxi, Shanghai, and Yunnan to cover north-south and east-west gradients), subsequently building province-specific models using the same analytical framework (Fig 5). For SARS-CoV-2, the simulations showed strong concordance with actual data in Yunnan (R^2^ = 0·73) and Shanghai (R^2^ = 0·72), but poorer performance in Heilongjiang (R^2^ = 0·49) and Shaanxi (R^2^ = 0·52). Regarding influenza A virus, the model achieved excellent consistency in Heilongjiang (R^2^ = 0·96), Shaanxi (R^2^ = 0·74), and Shanghai (R^2^ = 0·98), though performed suboptimally in Yunnan (R^2^ = 0·41). For RSV, the modeling demonstrated robust accuracy across all four provinces: Heilongjiang (R^2^ = 0·94), Shaanxi (R^2^ = 0·87), Shanghai (R^2^ = 0·69), and Yunnan (R^2^ = 0·90).

### External cohort validation

To validate the reliability of the model, we projected three-month positivity rate at both national and key regional levels and compared these predictions against respiratory pathogen surveillance data published by the China CDC and the National Center for Infectious Diseases. At the national level (Fig 4), the model’s predictions for October-December 2024 epidemic trends demonstrated: (1) Consistent in both trend and magnitude in forecasting SARS-CoV-2 and influenza A virus transmission patterns; (2) Consistent in trend but divergent in magnitude for human coronaviruses, RSV, rhinovirus and parainfluenza virus, and (3) Divergent in both trend and magnitude for Mycoplasma pneumoniae and Streptococcus pneumoniae.

For regional validation, we selected Shanghai, Yunnan, Shaanxi and Heilongjiang as representative areas. After environment-specific parameter adjustments (S3 Fig), the model’s predictions for October-December 2024 showed varying performance: (1) For SARS-CoV-2, high consistency with observations in Yunnan and Shanghai but divergent in Heilongjiang and Shaanxi; (2) For influenza A, high consistency in Heilongjiang, Shaanxi and Shanghai, but divergent in Yunnan; and (3) For RSV, high consistency across all four provinces. These geographically stratified results underscore both the model’s utility in routine surveillance and its limitations regarding exceptional epidemiological events.

### Model performance evaluation based on DISO comprehensive metric

We presented a comprehensive external validation of predictive models for influenza A, SARS-CoV-2, and RSV across four key regions (Shanghai, Yunnan, Shaanxi, and Heilongjiang), with comparative benchmarking against four widely used machine learning methods: random forest (RF), multiple linear regression (MLR), artificial neural network (ANN), and support vector machine (SVM), using an external validation cohort. Mean performance metrics including the correlation coefficient (CC), root mean square error (RMSE), and mean absolute error (MAE) were calculated for each model. To address potential inconsistencies among evaluation metrics when assessing the same pathogen, we employed the integrated DISO (Distance between Indices of Simulation and Observation) index, which quantifies multidimensional distances between predicted and observed outcomes [16,17]. The results demonstrate that the GBDT model achieved the lowest DISO values (0.12 in influenza A, 0.21 in SARS-CoV-2, 0.25 in RSV), confirming its superior predictive performance compared with alternative approaches (Table 1).

**Table 1:** Model performance evaluation based on DISO comprehensive metric.

Pathogens	AI models	CC	RMSE	MAE	DISO
Influenza A virus	GBDT	0.32	13.24	9.45	0.12
	RF	0.01	14.26	9.88	0.40
	MLR	0.19	14.58	11.04	0.50
	ANN	0.31	13.56	9.75	0.21
	SVM	0.02	13.02	9.45	0.32
SARS-CoV-2	GBDT	0.33	7.12	5.45	0.21
	RF	0.22	6.83	5.08	0.32
	MLR	0.31	8.01	7.04	0.45
	ANN	0.28	7.98	10.33	0.23
	SVM	0.3	9.12	4.65	0.34
RSV	GBDT	0.41	6.21	5.45	0.25
	RF	0.21	7.23	4.88	0.67
	MLR	0.19	8.83	6.04	0.45
	ANN	0.31	7.76	4.33	0.28
	SVM	0.31	9.12	4.65	0.32

Abbreviations: GBDT: Gradient Boosting Decision Tree; RF: Random Forest; MLR: Multiple Linear Regression; ANN: Artificial Neural Network; SVM: Support Vector Machine; RSV: Respiratory Syncytial Virus, CC: Correlation Coefficient, RMSE: Root Mean Square Error, MAE: Mean Absolute Error, DISO: Distance between Indices of Simulation and Observation.

## Discussion

This retrospective study of 115,406 respiratory samples (September 2022 and September 2024) characterized the temporal and seasonal patterns of 28 common community-acquired respiratory pathogens using tNGS and mNGS. Multifactorial nonlinear regression analysis revealed CO, HCHO, O3, sunshine hours, wind speed, and GDP as key drivers of positivity rate variations for most respiratory pathogens. Utilizing these critical determinants, we developed GBDT models to correlate nationwide monthly positivity rates of ten representative pathogens with climatic, environmental and economic variables. The GBDT approach outperformed alternative machine-learning methods, particularly for influenza A virus, SARS-CoV-2, and rhinovirus, and showed strong concordance with external cohort data.

Feature importance method based on mean Gini index reduction to confirmed CO, HCHO, O3, sunshine hours, wind speed, and GDP with high correlation with the prevalence of most respiratory pathogens nationwide (Fig 3), while demonstrating variations between northern and southern cities (S3 Fig). These findings align with Yongjian Zhu et al.’s observations of positive correlations between short-term air pollution indices and SARS-CoV-2 incidence in mainland China [18], Adriana Peci et al.’s negative correlations for both influenza A and B with relative humidity and temperature [19], Bin Chen et al.’s skin temperature, dew point, and southeast wind associations with SARS-CoV-2 transmission in Shanghai [20], Xu Bing et al.’s temperature, pressure, and vapor pressure links with six respiratory viruses [2].These cross-study validations underscore the robustness of environmental determinants in respiratory disease epidemiology while highlighting evolving climate-pathogen interactions in the pandemic era. A critical consideration arising from our findings is the distinction between predictive utility and biological causality. While our study identified climatic and environmental factors as important predictive indicators for the prevalence of various respiratory pathogens, the causal relationship between these geographical factors and respiratory disease transmission, as well as the underlying mechanisms, remain to be fully elucidated, which requires further investigation through mechanistic studies, longitudinal designs, and interventions that can disentangle these complex, potentially confounded relationships.

The GBDT model predicts respiratory-pathogen trends at national and key provincial/municipal levels by quantifying nonlinear driver effects and accommodating spatial heterogeneity. By auto-tuning parameters, it minimizes prediction error and delivers a unified risk-prediction framework for multiple respiratory pathogens across varied geographic settings. By integrating local geographic data, the model can be readily applied to predict respiratory‐disease trends in new regions. While our model demonstrates valuable capability for short-term forecasting of respiratory pathogen trends over the coming quarter, it is important to acknowledge its current limitations for long-term prediction. The present framework is not designed for, nor capable of, predicting epidemiological trends and pattern shifts over a one-year horizon or longer. Long-term transmission dynamics are influenced by complex and evolving factors such as shifts in population immunity, pathogen evolution, and long-term changes in social behavior, which are not captured by the meteorological and short-term environmental variables in our model. Therefore, attempting direct long-term prediction with limited historical data is not realistic. A more practical application pathway is to employ this model as a dynamic surveillance tool, continuously updating the training dataset with the latest surveillance data to enable rolling, iterative forecasts for the immediate future (e.g., the next quarter). To understand and anticipate fundamental shifts in epidemiological patterns across years or seasons, future work will require systematically collected data over much longer timeframes (e.g., encompassing full epidemic cycles) and potentially the development of novel modeling frameworks that integrate mechanistic insights with long-term drivers.

This retrospective study has several limitations and potential bias. First, the study is constrained by its two-year period (September 2022 to September 2024), a timeframe coinciding with a unique post-pandemic transition following the relaxation of China’s “Zero-COVID” policy. While this period was intentionally selected to re-evaluate climate-pathogen relationships altered by non-pharmaceutical interventions, its short duration and overlap with a major epidemiological recalibration period substantially limit the causal interpretation and generalizability of the identified climatic and socioeconomic drivers. The 2-year dataset also limited the interpretation for long inter-epidemic cycle pathogens. Long-term data spanning multiple typical seasons are needed to validate whether the observed relationships stabilize. Second, the data are derived exclusively from a single commercial laboratory network. While this provided a large, standardized dataset from over 1,880 centers, it may not be fully representative of the national population. Variations in healthcare-seeking behavior and access to specialized testing like NGS could introduce socioeconomic bias. This potential sampling bias may confound associations with predictors like GDP, as the observed correlations might reflect disparities in test utilization rather than true ecological relationships, thereby limiting the accurate assessment of regional prevalence differences and the influence of economic factors. Moreover, as a purely data-driven approach, it lacks mechanistic interpretability and may fail to anticipate atypical outbreak events that fall outside historical seasonal patterns [21,22]. Future work should focus on enhancing surveillance data quality, representativeness, and temporal span, and on integrating mechanistic insights with multi-scale modeling frameworks to develop robust, proactive early-warning systems.

In summary, this study developed a novel short-term prediction framework for respiratory pathogen diseases in China, empowered by heterogeneous multi-source big data and integrated artificial intelligence models. The proposed methodology demonstrates strong generalizability, enabling future applications for respiratory disease risk forecasting across most regions of mainland China. Furthermore, the framework exhibits potential for extrapolation to epidemiological assessments of other infectious diseases.

## Materials and methods

### Study design

We retrospectively collected 153,544 targeted next-generation sequencing (tNGS) or metagenomic next-generation sequencing (mNGS) data using respiratory samples (nasopharyngeal swabs, sputum, bronchoalveolar lavage fluid, pleural effusion, lung tissue) from patients with respiratory infections between September 2022 and September 2024. The data was provided by Dian Diagnostics (Hangzhou, China), a diagnostic company offering third-party testing services to more than 22,000 healthcare institutions across China. Demographic data (age, sex), infection site, sample type, detection method, and geographic information were also collected. The data analysis protocol was reviewed and approved by Huashan Institutional Review Board (KY2025–100, KY2024–668) and Di’an Diagnostics Institutional Review Board (D2024-P04-S01).

For the validation cohort, we retrospectively gathered monthly positive detection rates between October 2024 and December 2024 for SARS-CoV-2, influenza A virus, rhinovirus, Streptococcus pneumoniae, and Mycoplasma pneumoniae from two sources: a) Respiratory pathogen surveillance reports published by the Chinese Center for Disease Control and Prevention (China CDC) [23]; b) Tertiary surveillance data from the National Center for Infectious Diseases [24].

### NGS detection

Both tNGS and mNGS was performed by Dian Diagnostics (Hangzhou, China). For tNGS, 5 panels for respiratory pathogen detection were included for analysis including a common panel, tNGS100 panel, tNGS200 panel, tNGS500 panel and a Nanoseq panel. These 5 panels varied by pathogen spectrum but all covered common respiratory pathogens (S2 Table). Pathogen-specific targeted primers were used to enrich pathogenic microorganisms based on reverse transcription PCR and multiplex PCR amplification methods. Nucleic acids were extracted using a GenK Targeted Sequencing Library Preparation Kit (2102-02), tNGS was performed using a GenK Universal Targeted Enrichment Kit for Pathogenic Microorganisms (2062A). Rolling circle amplification was used to form DNA nano-balls. Human genomic sequences were then removed. Nonhuman sequences were aligned with target sequences to determine whether the suspected sample contains the corresponding pathogen. mNGS was performed with a standard clinical detection procedure and was divided by DNA + RNA, DNA-only and RNA-only detection, according to their pathogen spectrum.

### Community-acquired respiratory pathogens, co-infection and monthly positivity

Our analysis primarily focused on 28 major pathogens known to cause acute respiratory infections, including *Chlamydia psittaci*, influenza B virus, SARS-CoV-2, adenovirus, *Pseudomonas aeruginosa*, *Stenotrophomonas maltophilia*, *Legionella pneumophila*, *Enterococcus faecium*, human metapneumovirus, human bocavirus, *Haemophilus influenzae*, *Moraxella catarrhalis*, *Staphylococcus aureus*, Influenza A virus, respiratory syncytial virus (RSV), common coronaviruses, *Haemophilus parainfluenzae*, parainfluenza virus, *Bordetella parapertussis*, *Enterococcus faecalis*, *Mycoplasma pneumoniae*, *Chlamydia pneumoniae*, *Streptococcus pneumoniae*, *Klebsiella pneumoniae*, *Escherichia coli*, Influenza C virus, rhinovirus, *Acinetobacter baumannii*, and *Bordetella pertussis*. Fungi, tuberculosis, opportunistic pathogens, and colonizing bacteria were excluded from the current analysis.

Bacteria-virus coinfection was defined as a sample in which pathogenic bacteria and virus mentioned above were both detected. Bacteria coinfection was defined as a sample in which at least 2 pathogenic bacteria in the absence of any viruses. Bacteria mono-infection was defined as only one pathogenic bacterium without concurrent viral detection. Virus coinfection/mono-infection apply vice versa.

For each pathogen or infection pattern, we calculated the positive detection rate for a specific region and time period as follows: (Number of positive detections for a specific pathogen in a region during a period/ Total number of tests performed in that same region and period) × 100, where the number of tests must be > 30 and must include detection assays for the relevant pathogens.

### Geographic data source and management

Geographic data were obtained from the administrative division records of China’s Ministry of Civil Affairs. Through literature review and clinical expertise integration [25–28],we selected 12 geographic indicators spanning three categories: climate, air quality, and socioeconomic factors (Table 2). These multi-source geospatial data were linked to pathogen positivity rates through geographic coordinates and year-month temporal markers. For geospatial elements with incompatible spatiotemporal scales, spatial aggregation and spatiotemporal resampling methods were applied to achieve feature alignment across variables within unified geographic units, ultimately generating a complete Predictive Risk Dataset System (PRDS) for modeling purposes.

**Table 2:** Geographic data source.

Type	Factors	Source	URL
Region	Province	Ministry of Civil Affairs of the People’s Republic of China	http://xzqh.mca.gov.cn/map
Air Quality Index (AQI)	PM2.5, PM10	Ministry of Ecology and Environment of the People’s Republic of China	PM2.5/10 measurements were sourced from the official website of the Ministry of Ecology and Environment of China: https://www.mee.gov.cn
	SO2 (mean concentration)	European Space Agency https://www.tropomi.eu	COPERNICUS/S5P/NRTI/L3_SO2
	NO2 (mean concentration)		COPERNICUS/S5P/NRTI/L3_NO2
	HCHO (mean concentration)		COPERNICUS/S5P/NRTI/L3_HCHO
	O3 (mean concentration)		COPERNICUS/S5P/NRTI/L3_O3
	CO (mean concentration)		COPERNICUS/S5P/NRTI/L3_CO
Climate	Mean temperature Humidity Sunshine hours Wind speed	ECMWF daAGGR_DAILY/LAND5_ERA/taset	ECMWF/ERA5_LAND/DAILY_AGGR Muñoz Sabater, J., (2019)(30)
Economy	Gross Domestic Product (GDP)	National Bureau of Statistics of China	https://data.stats.gov.cn/easyquery.htm?cn=C01

#### Identification of geographic driving factors.

We employed the Gini coefficient to evaluate the contribution of various geographic parameters to respiratory pathogen positivity rates. A higher score indicates greater contribution of the parameter to positivity rate fluctuations, reflecting higher importance. The calculation formula is as follows:


$$\begin{array}{c} \text{Gini}=\text{1}-\sum_{i=\text{1}}p_{i}^{\text{2}} \end{array}$$ (1)

where $p_{i}$ represents the proportion of samples belonging to class i at the current node.

The feature importance based on Gini impurity reduction is calculated as follows:


$$\begin{array}{c} \Delta \text{Gini}={\text{Gini}}_{\text{F}}-(w_{\text{L}}\cdot {\text{Gini}}_{\text{L}}+w_{\text{R}}\cdot {\text{Gini}}_{\text{R}}) \end{array}$$ (2)

Where $w_{\text{L}}$, $w_{\text{R}}$ represent the proportion of samples in the left and right child nodes, respectively. The algorithm traverses all nodes in the complete tree constructed from the modeling data, summing the Gini impurity reduction contributed by each feature at every node - this sum constitutes the feature’s importance within a single tree. The model’s overall feature importance is obtained by averaging the importance values of each feature across all trees in the ensemble.

### Gradient boosting decision tree

We established initial models using GBDT (Gradient Boosting Decision Tree) by inputting the study dataset. At both national and regional levels, we selected different pathogen types to train and optimize models, obtaining distinct model parameters and weights. The trained models generated prediction outputs, which were subsequently validated against external cohorts (Fig 1).

Assume we have a dataset with sample pairs (x, y) for modeling, where the loss function is $L(y_{i},\gamma )$, with y being the true value and *F(x)* the predicted value. The process begins by constructing an initial model *F_0_(x)*, typically chosen as the constant value that minimizes the loss function (e.g., the mean):


$$\begin{array}{c} F_{\text{0}}(x)=\underset{\gamma }{argmin}\sum_{i=\text{1}}^{n}L(y_{i},\gamma ) \end{array}$$ (3)

After *m* rounds of iterative weak learner training, in each iteration the negative gradient (i.e., the current model’s prediction residuals) is calculated and used as the new target variable:


$$\begin{array}{c} r_{i(m)}={-[\frac{\partial L(y_{i},F(x_{i}))}{\partial F(x_{i})}]}_{F=F_{m-\text{1}}} \end{array}$$ (4)

For the j-th leaf node in the m-th iteration, its output value is calculated as follows (by minimizing the loss function to determine each leaf node’s optimal output value):


$$\begin{array}{c} \gamma_{jm}=\underset{\gamma }{argmin}\sum_{x_{i}\in R_{jm}}L(y_{i},F_{m-\text{1}}(x_{i})+\gamma ) \end{array}$$ (5)

Here, $\gamma_{jm}$ denotes the value of the *j*-th leaf node in the *m*-th iteration, $R_{jm}$ represents the sample set belonging to the *j*-th leaf node in the *m*-th iteration, *L* is the loss function, and $F_{m-\text{1}}\left(x_{i}\right)\;$indicates the predicted value from the previous iteration.

The model is updated according to Equation (6) after each training iteration:


$$\begin{array}{c} F_{m}(x)=F_{m-\text{1}}(x)+\nu \cdot \gamma_{m}h_{m}(x) \end{array}$$ (6)

where ν ∈ (0,1] is the learning rate that controls the contribution of each tree $h_{m}(x)$ to prevent overfitting, with $h_{m}(x)\;$defined as follows:


$$\begin{array}{c} h_{m}(x)=\sum_{j=\text{1}}^{J}\gamma_{jm}\cdot \text{1}\{x\in R_{jm}\} \end{array}$$ (7)

*J* denotes the number of terminal nodes (representing tree complexity), $R_{jm}$ corresponds to the region covered by the *j*-th terminal node in the *m*-th regression tree, $R_{jm}$ specifies the output value. $\text{1}\left\{x\in R_{jm}\right\}$ serves as an indicator function that evaluates to 1 when $x\in R_{jm}$ and 0 otherwise.

The comparative evaluation of multiple models (RF, MLR, ANN, etc.) was conducted using the comprehensive assessment metric DISO (Distance between Indices of Simulation and Observation), a comprehensive, multi-metric evaluation index. The theoretical foundation and computational methodology of DISO were derived from previous studies [16,17]. In the present study, DISO was employed to assess model simulation accuracy, with smaller DISO values indicating better model performance. The primary calculation is as follows (Fig 1):


$$\begin{array}{c} \begin{array}{c} {DISO}_{i}=\sqrt{{\left({nors}_{i}^{\text{1}}-{nors}_{\text{0}}^{\text{1}}\right)}^{\text{2}}+{\left({nors}_{i}^{\text{2}}-{nors}_{\text{0}}^{\text{2}}\right)}^{\text{2}}+\ldots +{\left({nors}_{i}^{n}-{nors}_{\text{0}}^{n}\right)}^{\text{2}}} \end{array} \end{array}$$ (8)

where *i* = 0, 1,..., *m*, with *m* representing the total number of modeling algorithms employed. The normalized model performance metrics (*nors*_*i*_^1^*, nors*_*i*_^2^*,..., nors*_*i*_^*n*^) incorporate the Correlation Coefficient (CC), Mean Absolute Error (MAE), and Root Mean Square Error (RMSE) as statistical indicators of model performance.


$$\begin{array}{c} \begin{array}{c} {DISO}_{i}=\sqrt{{\left({CC}_{i}-\text{1}\right)}^{\text{2}}+{\left(nor{MAE}_{i}-\text{0}\right)}^{\text{2}}+{\left({norRMSE}_{i}-\text{0}\right)}^{\text{2}}} \end{array} \end{array}$$ (9)

Where *i* = 0,1, …, m.

## Supporting information

S1 Fig Sample distribution per time (A) and provinces (B). (TIF)

S2 Fig Univariate linear correlation analysis between positivity rate and environmental factors. (TIF)

S3 Fig Feature importance of geographic factors on SARS-CoV-2, influenza A and RSV in Heilongjiang, Shannxi, Shanghai and Yunnan. (TIF)

S1 Table Baseline characteristics of respiratory infection patients enrolled between September 2022 and September 2024 in China. (XLSX)

S2 Table Respiratory pathogen spectrum by different detection methods. (XLSX)

S3 Table De-identified monthly pathogen positivity and geographic factors applied in this study. (XLSX)
