# Supplementary figures and images for: Artificial intelligence risk prediction model for common respiratory pathogens in China based on heterogeneous multi-source clinical and geographic data: A modeling study
Source: PLOS Digit Health. 2026 Jul 21;5(7):e0001553. doi: 10.1371/journal.pdig.0001553 (PMC13387545; doi:10.1371/journal.pdig.0001553)

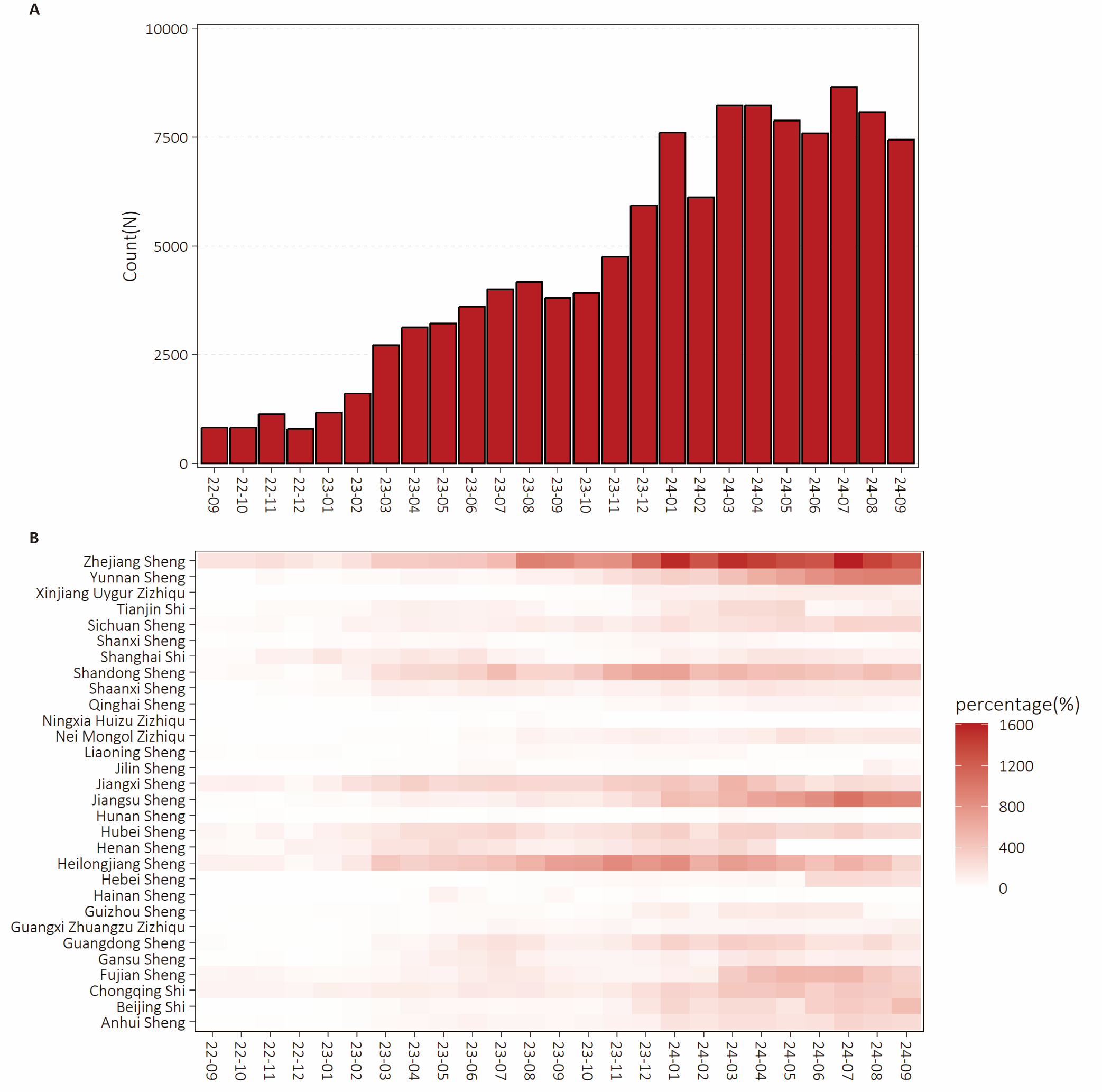

Supplement: S1 Fig — (TIF) [file pdig.0001553.s001.tif]

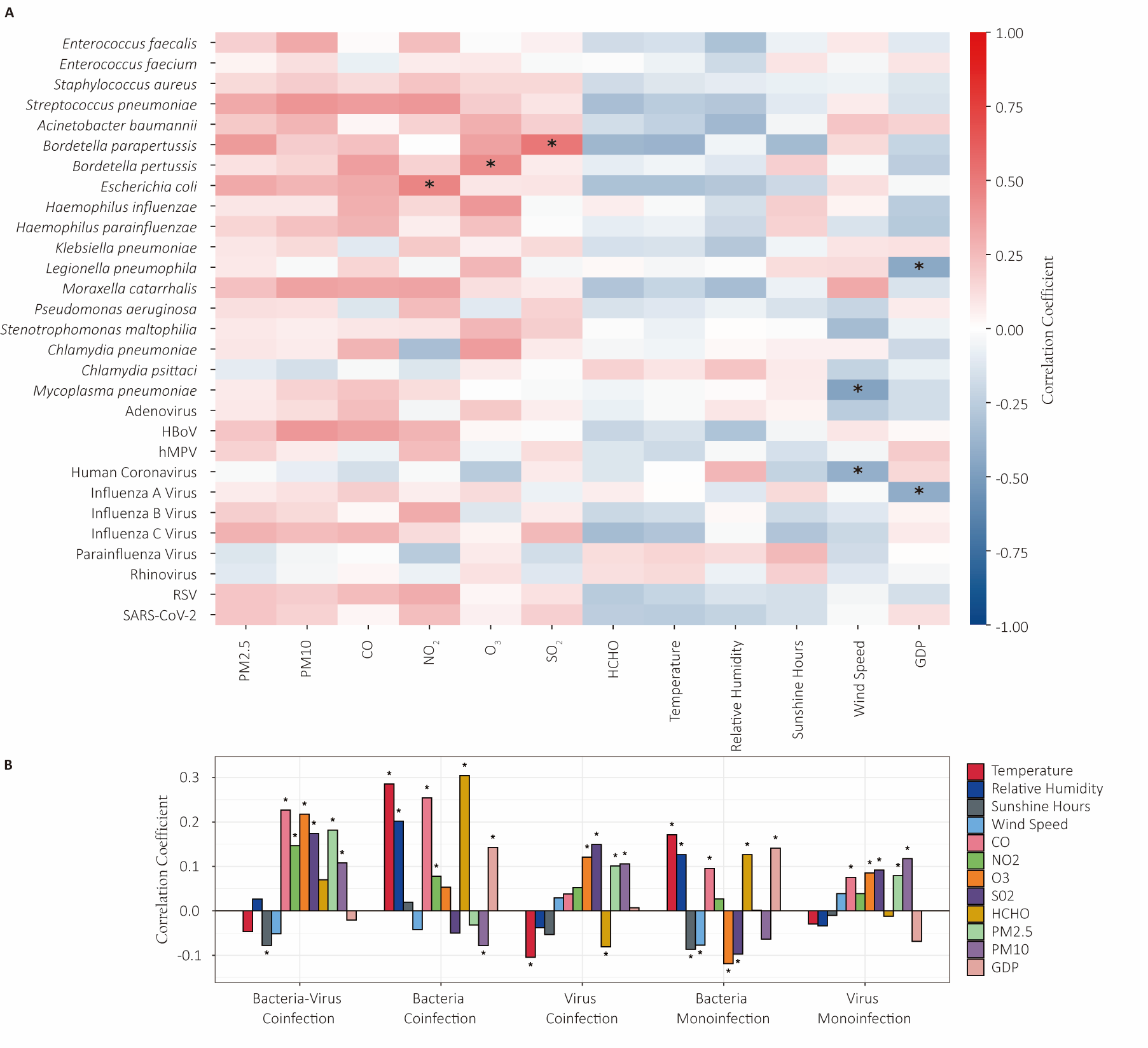

Supplement: S2 Fig — (TIF) [file pdig.0001553.s002.tif]

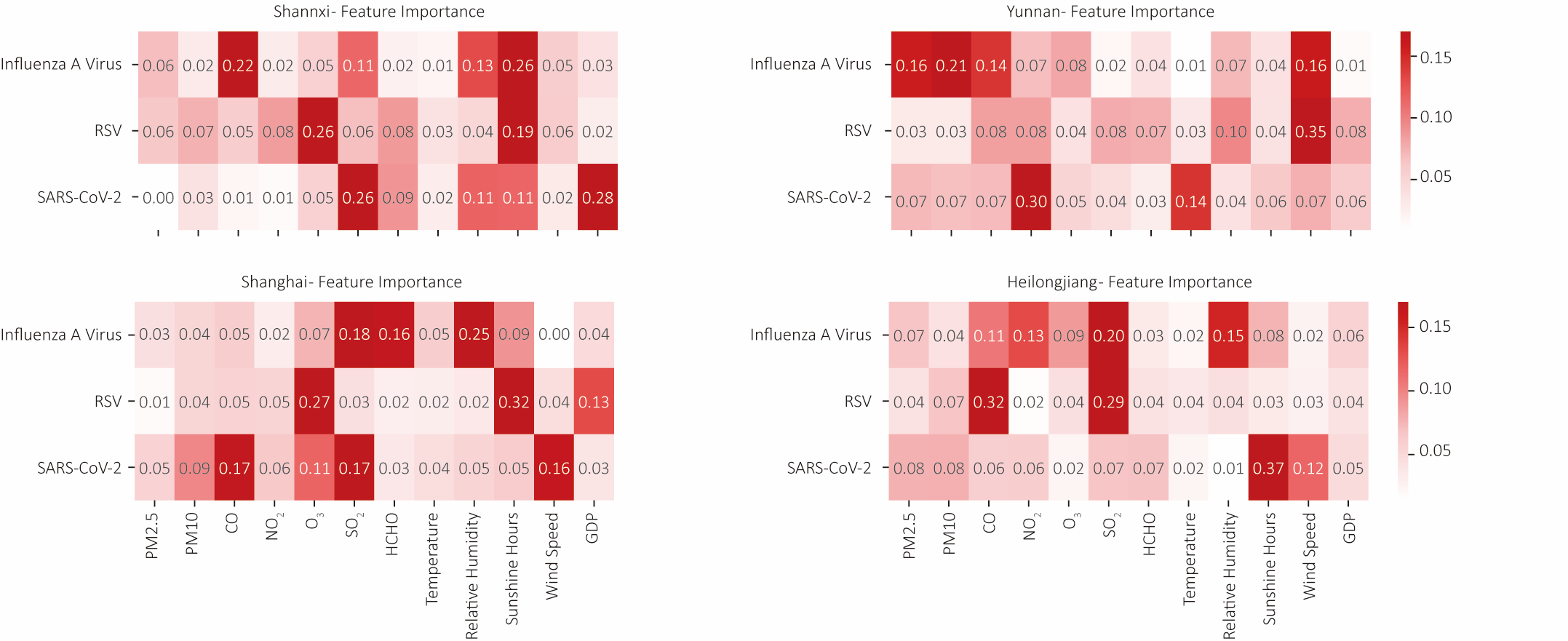

Supplement: S3 Fig — (TIF) [file pdig.0001553.s003.tif]
